# Oxidative stress impairs processive motility of the axonal transport motor KIF1A

**DOI:** 10.1016/j.jbc.2026.111471

**Published:** 2026-04-17

**Authors:** Adrien P. Chen, Himanshu Pandey, William O. Hancock

**Affiliations:** 1 Department of Biomedical Engineering, Pennsylvania State University, University Park, Pennsylvania, USA; 2 Department of Chemistry, Pennsylvania State University, University Park, Pennsylvania, USA

**Keywords:** kinesin, microtubule, oxidative stress, free radicals, neurodegenerative disease

## Abstract

The kinesin-3 family member, KIF1A is an essential motor protein that carries out intracellular transport in neurons. Previous work has established that: (1) intracellular transport can be impaired in neurodegenerative diseases such as Alzheimer's and Parkinson's; and (2) oxidative stress is elevated in neurodegenerative diseases and during aging. To date there has not been a systematic study of the effects of reactive oxygen species on kinesin motor proteins. We hypothesized that oxidative stress can damage kinesin, leading to decreased motility. To test our hypothesis, we treated KIF1A *in vitro* with varying concentrations of hydrogen peroxide (H_2_O_2_), a common reactive oxygen species, and characterized the impacts on KIF1A function. Pretreatment of KIF1A with H_2_O_2_ at concentrations of 1 mM and higher decreased motility in microtubule gliding assays. In single-molecule assays KIF1A was impacted in two ways: a fraction of motors moved with slowed velocity, while a fraction of motors moved only diffusively with no net directionality. Nonreducing SDS-PAGE of oxidized kinesin showed higher molecular weight bands, consistent with disulfide-bonded dimers and higher-order species. Treating oxidized motors with reducing agents reversed this cross-linking and partially restored motility. Replacing cysteine residues in the motor domain reduced the effects of moderate oxidation but did not prevent the severe degradation of motility at the highest H_2_O_2_ concentrations, indicating there is irreversible oxidative damage beyond only cysteine residues. Our results suggest that KIF1A can be impacted by oxidative stress and raise the possibility that oxidized KIF1A may be involved in the pathogenesis of neurodegenerative diseases.

KIF1A is a member of the kinesin-3 family that carries out intracellular transport in neurons, among other functions. It is among the fastest and most processive of all kinesins, reaching speeds up to 1500 nm/s and run lengths of microns (1). Consistent with its important role in axonal transport in motor neurons, a family of neurological diseases, KIF1A-associated neurological disorder (KAND), has been linked to mutations in KIF1A. KAND affects children and adolescents, and involves seizures, cerebellar atrophy, cognitive degeneration, and vision loss, among other symptoms (2).

In cells, oxidative stress is mediated by reactive oxygen species (ROS), which are reactive due to their lone electron radical and result in them removing electrons from (oxidizing) nearby molecules. Low levels of ROS are healthy for cell function and play roles in physiological signaling and the immune system (3). However, high concentrations of ROS can oxidize DNA, lipids and proteins, causing damage to those macromolecules and even cell death (4). Oxidative stress is a hallmark of aging and has been linked to neurodegenerative diseases such as Alzheimer's (5, 6, 7), Parkinson's (8), and amyotrophic lateral sclerosis (9).

When ROS come into contact with proteins, they can oxidize specific amino acids, leading to oxidative posttranslational modifications (oxPTMs) (10). Of the 20 amino acids, cysteine is the most susceptible to oxPTM and has multiple oxPTM forms, including a sulfenic group (S-OH), sulfinic acid (SO_2_H), sulfonic acid (SO_3_H), S-nitrosylation, and S-glutathionylation (11, 12). Although the sulfenic group can be reversed with reducing agents such as DTT, the sulfinic and sulfonic acids oxPTM are essentially irreversible (13). Another common cysteine posttranslational modification is the formation of a disulfide bond (11). While disulfide bonds form under normal conditions, excessive disulfide bond formation is associated with neurological diseases such as tauopathies (14). In addition to cysteine, methionine, proline, tyrosine, phenylalanine, tryptophan, histidine, lysine, and arginine can all be covalently modified by ROS (15, 16, 17, 18).

Previous studies have found that oxidative stress can alter the functional properties of cytoskeletal filaments and motor proteins. For instance, S-nitrosylation of Cys374 in actin causes actin filament depolymerization (19) and oxidation of microtubules with H_2_O_2_ causes lattice damage and alters dynamics by increasing catastrophe and rescue frequencies (20, 21). Met394 on cardiac myosin is a redox-sensitive residue that can be reversibly oxidized to methionine sulfoxide, resulting in a suppression of the myosin ATPase activity (22, 23). In the plant *Kalanchoe pinnata*, it was shown that kinesin motors can be S-nitrosylated by the free radical nitric oxide (NO), but the effect of that modification on the motility was not characterized (24). It is currently unknown whether dynein is sensitive to oxidative posttranslational modifications, but exposing neurons in culture to H_2_O_2_ result in diminished axonal transport in both directions, consistent with disruption of both kinesin and dynein motility (25).

Due to the role of protein oxidation in aging and neurodegenerative disease and the importance of kinesin-driven axonal transport for neuronal function, we speculated that oxidative stress may directly impact kinesin motor protein function. The goal of the present study is to answer the question: How does oxidation impact KIF1A function? KIF1A is of particular interest due to its role in KAND. Using multimotor and single-motor assays *in vitro*, we find that oxidative stress by H_2_O_2_ diminishes the motile properties of KIF1A and cross-links dimers through their motor domains, inactivating them. In contrast, similar treatment of taxol-stabilized microtubules had no measurable effect on KIF1A motility. This work supports a model in which oxidative stress in aging or neurodegenerative disease damages neurons by diminishing kinesin-driven axonal transport.

## Results

To investigate the impact of oxidative damage on KIF1A function, we employed a previously characterized KIF1A dimer consisting of the head and neck linker regions (1–368) of *Rattus* *norvegicus* KIF1A followed by a kinesin-1 dimerization domain to ensure stable dimerization (345–560 of *Drosophila melanogaster* KHC), and a green fluorescent protein (GFP) and His_6_ tag (26). Motors were expressed in bacteria and purified by Ni column chromatography (26). Purified KIF1A proteins were oxidized by incubating with varying concentrations of hydrogen peroxide, and the effects of oxidation were characterized *in vitro* by multimotor and single-motor motility assays (27, 28, 29, 30).

### Oxidation of KIF1A by H_2_O_2_ decreases velocity in the microtubule gliding assay

**Figure 1:**
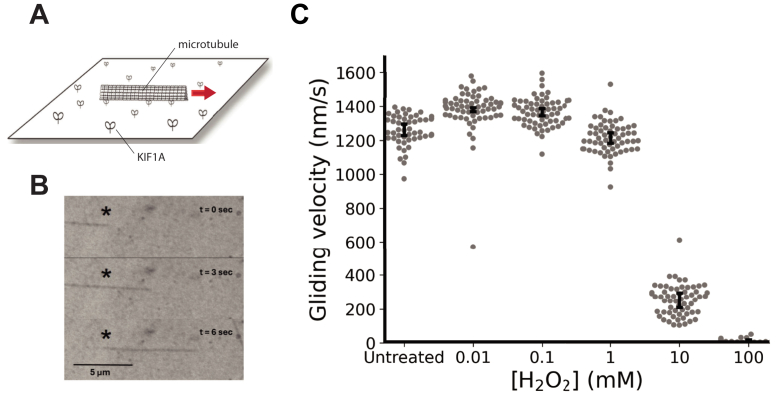
**H_2_O_2_ treatment of KIF1A reduces microtubule velocity in multimotor gliding assay.***A*, schematic of gliding assay. *B*, successive images from a movie of microtubule gliding, imaged by interference reflection microscopy. *C*, microtubule gliding velocity following treatment of motors with increasing concentrations H_2_O_2_. Motors (2.2 μM) were treated for 30 min with H_2_O_2_ and then diluted to 100 nM for gliding assays. Each point represents average and SEM of N = 60 microtubules combined from three independent days. H_2_O_2_, hydrogen peroxide.

To determine the effect of oxidation on the functional properties of teams of motors, we used the *in vitro* gliding assay in which motors are adsorbed to the surface and microtubules are observed gliding along the surface (Fig. 1*A*). Before adsorbing motors to the surface, 2.2 μM KIF1A was reacted with varying concentrations (10 μM–100 mM) of H_2_O_2_ for 30 min at room temperature in BRB80 buffer. The motor solutions were then diluted to 100 nM in buffer containing casein and taxol (Experimental procedures) and introduced into the flow cell. Following motor adsorption, a solution of taxol-stabilized microtubules in 2 mM ATP was introduced and microtubule gliding velocities were measured by interference reflection microscopy (Fig. 1*B*). We observed a clear decrease in microtubule velocity, starting at 1 mM H_2_O_2_ with a half-maximal effect between 1 mM and 10 mM H_2_O_2_. Subsequently, 100 mM H_2_O_2_ completely abolished movement (Fig. 1*C*).

### KIF1A oxidation slows motor velocities and reduces the fraction of processive motors in a single-molecule assay

**Figure 2:**
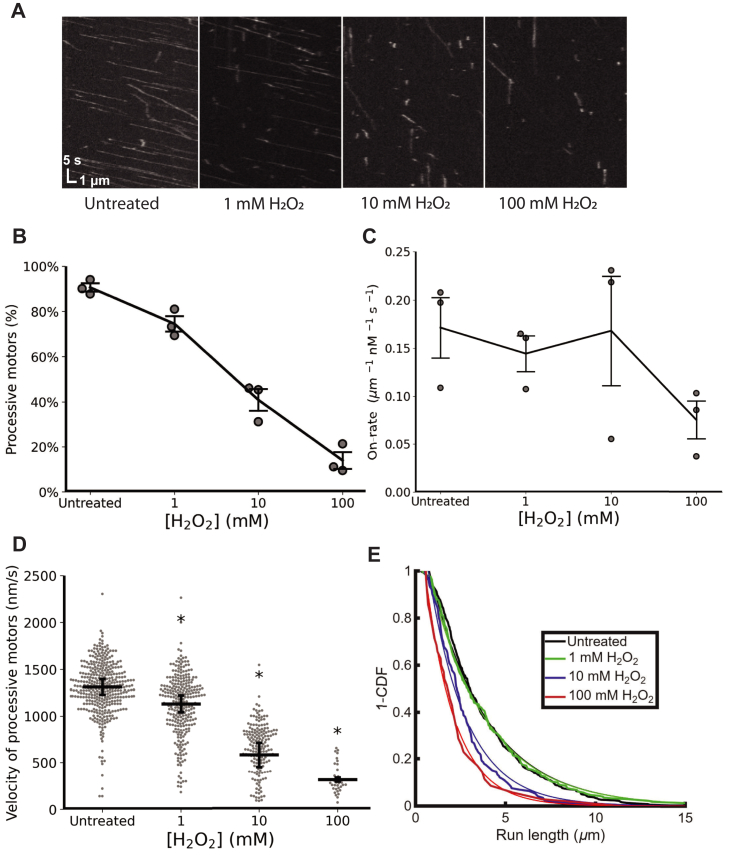
**H_2_O_2_ reduces KIF1A processivity and velocity in single-molecule assay.***A*, single-molecule kymographs of KIF1A in 2 mM ATP at different H_2_O_2_ concentrations. Motors (2.2 μM) were treated for 30 min with H_2_O_2_ and then diluted to 300 pM for single-molecule assay. *B*, fraction of processive motors. *C*, single-molecule motor landing rate, including both processive and nonprocessive motors. Each point represents an independent flow cell from at least two experimental days; error bars are SEM. *D*, velocity of processive motors. Treated groups were compared individually to the untreated control using a two-tailed unpaired student *t-*test with Bonferroni correction. *p-*values (where ∗ represents *p* < 0.05) were 0.00083, 0.026, and 0.013 for 1, 10, and 100 mM H_2_O_2_, respectively. Error bars represent SEM from 1 to 3 fields each from 3 flow cells, where each flow cell contained 60 to 230 kinesin landing events. *E*, run length of processive motors plotted as 1–cumulative distribution function and fit with a single exponential. Run lengths (fit and 95% confidence intervals) were as follows: Untreated: 3.21 μm [3.14, 3.28]; 1 mM H_2_O_2_: 3.22 μm [3.17, 3.27]; 10 mM H_2_O_2_: 2.11 μm [2.05, 2.18]; 100 mM H_2_O_2_: 1.64 μm [1.56, 1.73]. H_2_O_2_, hydrogen peroxide.

To more precisely determine the effect of oxidative stress on KIF1A mechanochemistry, we turned to the single-molecule assay in which fluorescent motors are visualized moving along immobilized taxol-stabilized microtubules. KIF1A samples were treated with H_2_O_2_ similar to gliding assays, diluted from 2.2 μM to 300 pM, and introduced into a flow cell containing immobilized microtubules (see Experimental procedures for details). Microtubule positions were identified by interference reflection microscopy, and the movement of GFP-labeled motors was visualized by total internal reflection fluorescence (TIRF) microscopy at 5 frames/s. Kymographs were generated from the movies and used to characterize motor landing rate and processive velocity (Fig. 2*A*).

The first observation was that the percentage of processive motors, defined as motors that moved at 100 nm/s or faster, decreased monotonically with [H_2_O_2_] (Fig. 2*B*). Interestingly, a small fraction of motors retained their activity at 100 mM H_2_O_2_, indicating the lack of motility at this concentration in the multimotor gliding assay (Fig. 1*C*) was likely due to immotile motors locking the microtubule on the surface. The second observation was that oxidative damage had a negligible effect on the motor on-rate up to 10 mM H_2_O_2_, though it fell at 100 mM H_2_O_2_ (Fig. 2*C*). In contrast, both the velocity and the run length of the remaining processive motors were strongly reduced at elevated H_2_O_2_ (Fig. 2, *D* and *E*). Thus, oxidative damage does not simply generate a population of completely inactive motors that do not interact with microtubules. Instead, oxidative damage impacts the mechanochemical function of KIF1A, resulting in motors that bind microtubules but walk more slowly and have shorter processive runs, or bind microtubules without moving.

### Treating microtubules with H_2_O_2_ does not affect the speed of KIF1A walking

**Figure 3:**
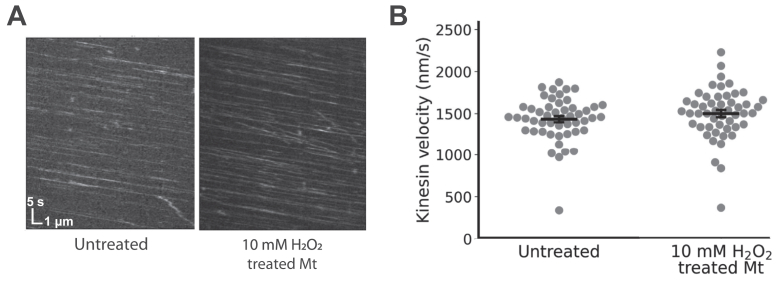
**Treatment of microtubules with 10 mM H_2_O_2_ does not affect KIF1A velocity.***A*, kymographs of KIF1A moving along untreated microtubules and microtubules incubated in 10 mM H_2_O_2_ for 30 min. *B*, single-molecule KIF1A velocity on control and H_2_O_2__-_treated microtubules. Error bars represent SEM (N = 50 binding events). Mean speeds are not significantly different (*p* = 0.14) by an unpaired Student's *t-*test. H_2_O_2_, hydrogen peroxide.

In principle, disease-induced oxidative stress in neurons could impact axonal transport through either an effect on the motors themselves, an effect on their microtubule track, or both. A previous electron microscopy study found that hydrogen peroxide can damage microtubules, creating holes in the lattice and increasing microtubule curvature (20); however, whether this impacts kinesin motility was not investigated in that work. To test whether oxidative stress from H_2_O_2_ damages microtubules in a way that impacts KIF1A stepping, we treated microtubules with H_2_O_2_ and measured the resulting KIF1A motility. Microtubules were treated with 10 mM H_2_O_2_ for 30 min while the microtubules were fixed on the glass slide in a flow cell. The H_2_O_2_ was then washed out and untreated KIF1A motors were flushed in and visualized (Fig. 3*A*). Notably, the KIF1A velocity did not significantly differ between control microtubules (1434 ± 37 nm/s; mean ± SEM, N = 50 events) and H_2_O_2__-_treated microtubules (1497 ± 43 nm/s; N = 50) (Fig. 3*B*). Thus, in contrast to its strong effect on KIF1A, treatment of microtubules with H_2_O_2_ had little effect on motility. This lack of effect also confirms that the loss of motility we observed when KIF1A was treated was due to motor oxidation, rather than any oxidative damage to the microtubules.

### Oxidative damage is partially reversible

**Figure 4:**
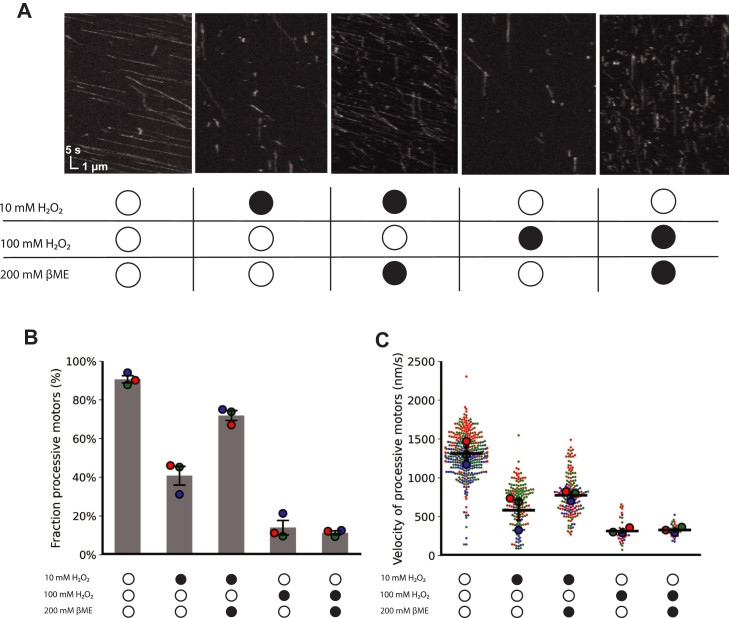
**βME treatment recovers the function KIF1A oxidized by 10 mM H_2_O_2_ but not 100 mM H_2_O_2_.***A*, single-molecule kymographs of KIF1A in 2 mM ATP following treatment with 10 mM and 100 mM H_2_O_2_, with and without subsequent treatment with 200 mM βME. Untreated, 10 mM H_2_O_2_, and 100 mM H_2_O_2_ kymographs are reproduced from Figure 2A for comparison. *B*, fraction of processive motors following treatments, mean ± SEM from N = 3 different flow cells. *C*, velocity of processive motors following treatments. Error bars represent standard error from N = 3 flow cells (larger points), each of which contained 60 to 230 kinesin landing events (smaller points). Colors represent different days. In *panelsB* and *C*, data for untreated, 10 mM H_2_O_2__,_ and 100 mM H_2_O_2_ are the same as presented in Figure 2. βME, 2-Mercaptoethanol; H_2_O_2_, hydrogen peroxide.

To test the reversibility of the oxidative damage, we treated oxidized KIF1A motors for 15 min with 200 mM of the reducing agent 2-Mercaptoethanol (βME). For motors treated with 10 mM H_2_O_2_, motility was substantially restored by βME—the fraction of processive motors rose from 41% up to 74% and the mean motor velocity increased from 584 nm/s to 1332 nm/s (Fig. 4). However, for motors treated with 100 mM H_2_O_2_, which severely impaired motor function, βME treatment had little effect. Thus, oxidation of KIF1A by 10 mM H_2_O_2_ causes both reversible and irreversible damage in a dose-dependent manner.

### Oxidation does not affect weak electrostatic association of KIF1A with microtubules

**Figure 5:**
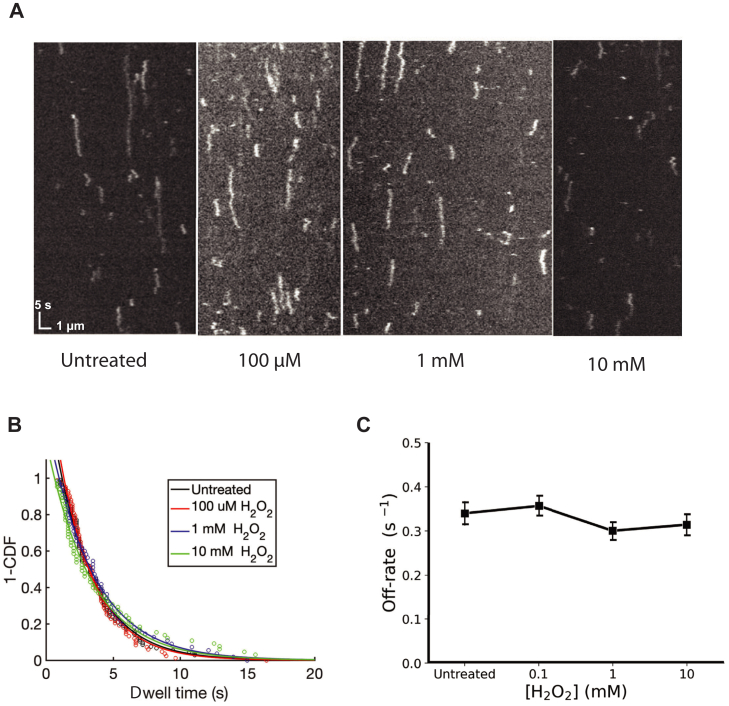
**KIF1A off-rate in ADP is not affected by moderate H_2_O_2_ treatment.***A*, kymographs of KIF1A in 1 mM ADP following motor treatment at different H_2_O_2_ concentrations. *B*, KIF1A dwell time distributions. *C*, KIF1A off-rate in ADP measured in single-molecule assay from fit to dwell time distributions. Error bars represent 95% confidence intervals of fits. N = 55 to 110 binding events per condition. H_2_O_2_, hydrogen peroxide.

Having characterized the motility of oxidized KIF1A in ATP, we next analyzed the microtubule off-rate of KIF1A in its weak-binding ADP state. KIF1A is notable in its strong electrostatic interaction with microtubules, which is mediated through the interaction of the positively charged motor Loop 12 with the negatively charged C-terminal tail of tubulin (31, 32). This electrostatic interaction can be evaluated by analyzing KIF1A single-molecule behavior in 1 mM ADP, which puts the motor in its weak-binding state. As seen on the raw kymograph images in Figure 5*A*, KIF1A motors in ADP diffused along microtubules for multiple seconds across a range of [H_2_O_2_]. The KIF1A off-rate from the microtubule was evaluated by fitting an exponential function to the distribution of single-molecule dwell times (Fig. 5*B*). Comparison of the off rates shows no significant difference between the control and the H_2_O_2__-_treated groups (Fig. 5C).

The lack of change in the KIF1A off-rate in its weak binding state suggests that the microtubule binding interface is not significantly affected by H_2_O_2_. Thus, the decrease in processivity and velocity in ATP are likely not due to altered microtubule binding but instead are due to other disruptions of the mechanochemical cycle.

### Oxidation causes dimerization of KIF1A *via* reversible disulfide bonds

**Figure 6:**
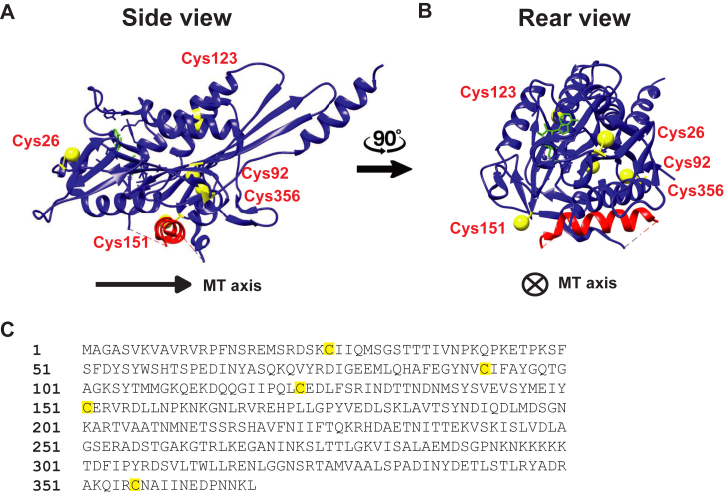
**Location of cysteines in KIF1A motor domain.***A*, rear view and *B*, side view of KIF1A motor domain (PDB ID: 7EO9) showing locations of cysteine residues. α-helix 4 that sits on the microtubule is highlighted in *red*, and helix to the *right* in A is the proximal neck-coil of KIF1A (α-helix 7). Note that C92 is buried, C26, C123, and C151 are solvent-exposed, and C356 is in the neck linker domain. *C*, sequence of the KIF1A motor and neck linker domain showing cysteine residues highlighted in *yellow*. PDB, Protein Data Bank.

**Figure 7:**
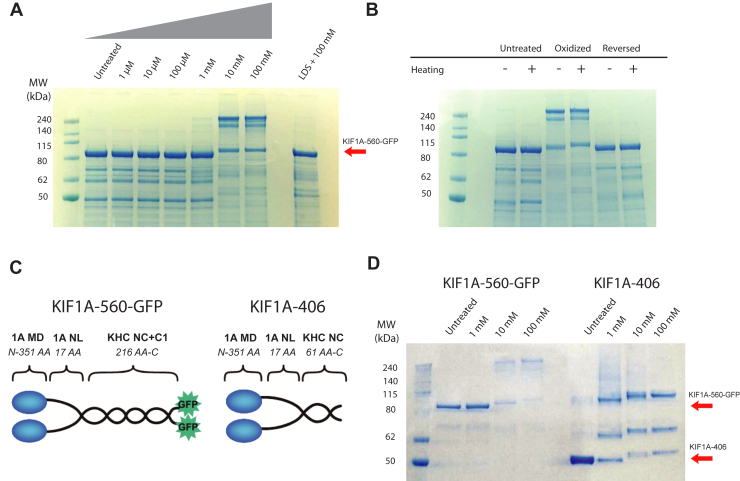
**Treating KIF1A with H_2_O_2_ results in reversible disulfide bond formation.***A*, KIF1 motors treated with increasing concentrations of H_2_O_2_. Higher molecular weight bands, consistent with disulfide cross-linking are observed at 1 mM H_2_O_2_ and above. The sample in the last lane was first denatured with LDS before H_2_O_2_ treatment, showing that disulfide crosslinking requires physical proximity of the two motors in the dimer. *B*, KIF1A crosslinking formation can be reversed with DTT treatment but not by heat alone. Control group, treated group (100 mM H_2_O_2_) and rescued group (100 mM H_2_O_2_ followed by 200 mM DTT) are shown without a heating step (−) and with an incubation at 100 °C for 5 min following treatment (+). *C*, schematic of KIF1A-560-GFP construct used for all motility assays and previous gels, and KIF1A-406 construct used to test cross-linking in distal coiled-coil. *D*, LDS-PAGE gel showing that dimer forms *via* oxidation in both the KIF1A-560-GFP and KIF1A-406 construct, consistent with disulfide cross-linking occurring between the two motor domains in a KIF1A dimer. LDS, lithium dodecyl sulfate; H_2_O_2_, hydrogen peroxide.

We next sought to determine the specific chemical modifications resulting from H_2_O_2_ treatment. Because KIF1A function could be partially restored by treating with reducing agents, the most likely modifications are reversible S-nitrosylation, S-glutathionylation, S-acetylation, or disulfide bond formation of cysteine residues (13). There are five cysteine residues in the KIF1A motor domain (Fig. 6). We first investigated whether intermolecular disulfide bonds are being formed in oxidized KIF1A by running treated motors on nonreducing SDS-PAGE gels and determining whether bands corresponding to KIF1A are seen at double their molecular weight. KIF1A motors were treated with H_2_O_2_ as before, and then the reactions were quenched by denaturing the protein with ∼2.5% (w/v) lithium dodecyl sulfate (LDS). In the control lane (untreated), we observed a band around 90 kDa, corresponding to the molecular weight of a denatured KIF1A monomer (93.8 kDa) (Fig. 7*A*). In H_2_O_2_-treated samples, bands around 180 kDa appeared, consistent with a cross-linked dimer, and around 270 kDa, consistent with a trimer. These higher molecular weight bands first appeared in the 1 mM group, where deterioration of KIF1A function was observed and became prominent with 10 mM and 100 mM H_2_O_2_ treatment (Fig. 7*A*). The simplest interpretation is that cross-linked dimers result from intramolecular disulfide bonds in KIF1A dimers. A tentative interpretation of trimers is that intermolecular disulfide bonds are formed between a disulfide-crosslinked dimer and a non-crosslinked dimer, resulting in a crosslinked trimer upon denaturation. Since multiple disulfide bonds are required to create trimers, this implies that more than one cysteine is involved in the cross-linking. In all cases, there was a residual band near the monomer molecular weight, which may imply intrachain crosslinking such as between the head and the coiled-coil or GFP domains. Importantly, no KIF1A dimers were formed if proteins were denatured before the oxidation treatment (Fig. 7*A*, last lane on the gel), indicating that physical proximity through coiled-coil dimerization is required for disulfide cross-linking under our conditions.

To test the reversibility of disulfide bond formation, motors were treated with 100 mM H_2_O_2_ followed by treatment with 200 mM of the reducing agent dithiothreitol (DTT) (Fig. 7*B*). The high molecular bands disappeared in the DTT-reversed group, showing that the disulfide bonds are reversible. Each group was also exposed to 5 min heating at 100 °C after the oxidation and LDS denaturation to test whether cross-linking could be removed by heat alone. We found that heating alone did not reverse disulfide cross-linking. The fact that the H_2_O_2_ concentration dependence of disulfide cross-linking in Figure 7*A* parallels the loss of motility in Figures 1*C* and 2*B* provides evidence that disulfide cross-linking is responsible for the reversible loss of KIF1A motility following H_2_O_2_ treatment.

Based on the similar sensitivity of cross-linking and motility to oxidation, the most obvious interpretation is that the heads are cross-linked through cysteine residues in the motor domain or neck linker, preventing stepping. Because engineered kinesins are quite tolerant of swapping and cross-linking coiled-coil domains (26, 29, 33), it is less likely that cross-linking in the coiled-coil causes inhibition of motility. However, to confirm that cross-linking was not solely due to cysteine residues in the coiled-coil domain, we repeated the treatment on a construct, KIF1A-406, that lacks the distal coiled-coil and contains no cysteines in its shorter neck coiled-coil domain (Fig. 7*C* (26)). Oxidation of KIF1A-406 by H_2_O_2_ resulted in higher molecular weight species (Fig. 7*D*), consistent with cross-linking occurring through the motor domains.

### Mutating cysteines in KIF1A motor domain resulted in a slower motors with partial resistance to oxidation

**Figure 8:**
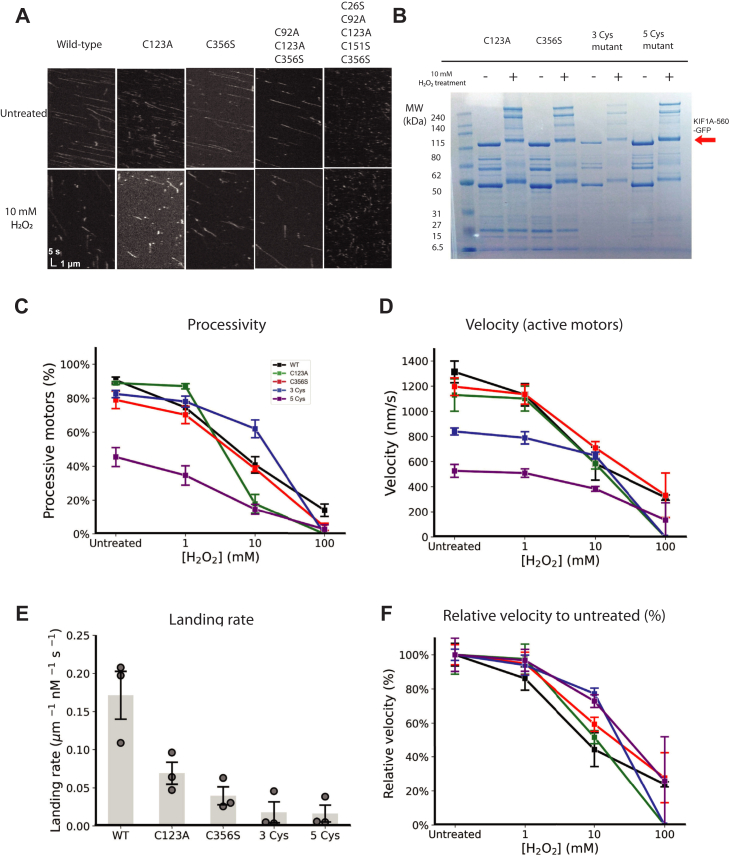
**Characterization of KIF1A cysteine mutants following H_2_O_2_ treatment.***A*, single-molecule kymographs of KIF1A cysteine mutants, untreated (*top*) and following 30 min treatment with 10 mM H_2_O_2_ (*bottom*). *B*, nonreducing SDS-PAGE gel of KIF1A Cys mutants showing cross-linking of all mutants tested. Monomeric KIF1A bands are shown with an *arrow*. *C*, fraction of processive motors at varying [H_2_O_2_]. *D*, velocity of processive motors. *E*, landing rate for WT and cysteine-mutated motors before H_2_O_2_ treatment. *F*, motor velocities as a function of [H_2_O_2_], normalized to the velocity of each motor before treatment. When comparing normalized velocities at 10 mM H_2_O_2_, both the 3 Cys and 5 Cys mutants were greater than control with *p* < 0.05 by one-tail paired *t*-test. Error bars in *D* and *F* represent SEM with between 60 and 100 binding events across three replicas for each motor. Error bars in C and E represent SEM from three replicas. Kymographs for WT untreated and 10 mM H_2_O_2_ in *panel A* and all WT data in *panels C-E* are reproduced from Figure 2. H_2_O_2_, hydrogen peroxide.

To test the hypothesis that H_2_O_2_ alters KIF1A function by modifying cysteine residues in the motor domain, we systematically eliminated some or all of the five cysteine residues in the KIF1A motor domain (Fig. 6). The following four mutants were created: single cysteine mutants C123A and C356A, triple-cysteine mutant C92A_C123A_C356S, and penta-cysteine mutant C26S_C92A_C123A_C151S_C356S. All mutants were motile and their motility was diminished following H_2_O_2_ treatment (Fig. 8*A*). To determine whether cysteine removal prevented disulfide cross-linking, we treated the motors with 10 mM H_2_O_2_ and ran them on a nonreducing SDS-PAGE gel (Fig. 8*B*). Higher molecular weight bands, corresponding to dimer and trimers, were present in all four mutants, even the penta-cysteine mutant. We attribute this cross-linking to cysteine residues in the KIF1A coiled-coil (Cys465) or the GFP tag (Cys633 and Cys655). Previous work showed that crosslinking kinesin-1 through cysteines in the coiled-coil region had no effect on motor function (33). Combining these cross-linking results with the finding that K406, which contains no cysteines distal to the motor domain, is cross-linked by 10 mM H_2_O_2_ (Fig. 7*D*), we conclude that crosslinking occurs both in the motor domain and the coiled-coil/GFP region in our construct.

We next characterized the functional properties of the cysteine mutants by analyzing their motility in the single-molecule assay. The two single-Cys mutants behaved similarly to WT, with negligible change in the fraction of processive motors or velocity, although their landing rates were diminished. In contrast, the untreated triple and penta mutants had slower velocities and lower landing rates than WT (Fig. 8, *C*–*E*). Thus, removing cysteines did diminish motor performance, but the motors remained sufficiently functional to test the impact of H_2_O_2_ treatment. When motors were treated with increasing concentrations of H_2_O_2_, the fraction of processive motors and the single-molecule velocities all fell qualitatively similarly to the WT control (Fig. 8, *C* and *D*). Hence, removing all of the cysteines in the motor domain does not protect KIF1A against oxidative damage by H_2_O_2_. To assess the relative impact of oxidation, we normalized the velocity of each motor to its value before treatment (Fig. 8*F*). Interestingly, at 1 mM and 10 mM H_2_O_2_, the relative decrease in velocity was smaller for both the triple and penta-cysteine mutants than WT KIF1A. Similarly, at 10 mM H_2_O_2_ the fraction of processive motors was greater for the triple mutant than WT. Thus, removing cysteines in the motor domain partially protected KIF1A from oxidative damage at moderate H_2_O_2_ concentrations. At 100 mM H_2_O_2_, however, the motility was severely diminished for all mutants tested. From these results, we conclude that H_2_O_2_ oxidation of KIF1A *in vitro* has irreversible effects on KIF1A function that goes beyond cross-linking and modification of cysteine residues.

## Discussion

Although the effects of oxidative stress have been characterized on microtubules (20), actin (19) and myosin (22, 23), its effect on kinesin motor proteins remains relatively unexplored. We show here that H_2_O_2_ mediated oxidation of the kinesin-3 motor KIF1A reduces the fraction of processive motors and reduces the velocity of the remaining active motors, while minimally affecting the microtubule on-rate. Gel analysis suggested that motor dimers are being reversibly cross-linked by disulfide bond formation, inactivating them. However, removal of cysteines in the KIF1A motor domain did not prevent oxidative damage and, following maximal H_2_O_2_ treatments, reducing agents did not restore KIF1A motility. Thus, oxidation has complex and graded effects on KIF1A function, suggesting that in neurons chronic oxidative stress associated with aging or neurodegenerative diseases could negatively impact axonal transport.

### Oxidative stress damages KIF1A mechanochemistry rather than inhibiting microtubule binding

The clear decrease in microtubule gliding velocity in the multimotor gliding assay was the first indication that oxidation alters KIF1A in a graded, rather than all-or-none manner. Microtubule gliding velocity has been shown to depend weakly on the density of motors adsorbed to the glass surface (34, 35); thus, if oxidation completely inactivated the motors and prevented microtubule binding, we would not expect to see any decrease in the gliding velocity. By using the more precise single-molecule assay, we were able to more specifically define functional changes resulting from H_2_O_2_ oxidation: (1) it reduced the fraction of processive motors (Fig. 2*B*), and (2) it reduced the velocity of the remaining processive motors (Fig. 2*C*). The concentration-dependent reduction in velocity was greater in the multimotor gliding assay than in the single-molecule assay, consistent with the slowest subpopulation of motors oxidized determining the overall velocity. Following H_2_O_2_ treatment, the majority of the nonprocessive motors in the single-molecule assay were statically stuck to the microtubule rather than diffusing on the microtubule (compare static motors in ATP (vertical lines in Fig. 2*A*) with diffusive motors in ADP; Fig. 5*A*). We hypothesize that it is these stuck motors that limit the microtubule velocities in multimotor gliding assays.

The finding that the KIF1A landing rate in ATP (Fig. 2*C*) and the off-rate in ADP (Fig. 5*C*) were unchanged by moderate (10 mM) H_2_O_2_ treatment suggests that oxidative modifications do not primarily target the microtubule binding interface of KIF1A. The KIF1A microtubule binding site includes the canonical switch-II/α-4 helix and loop-11 regions (36, 37), along with the positively charged K-loop/loop-12, which interacts with the negatively charged C-terminal tail of tubulin, and is thought to mediate the diffusion of KIF1A on microtubules in ADP (31, 32, 38). The motility results suggest that oxidative modifications are most likely localized on other exposed loops, near the nucleotide binding site, or in the force-generating neck linker domain.

### Oxidative stress induces both reversible and irreversible damage to KIF1A

Interestingly, the loss of motility following moderate oxidation was partially rescued by treating oxidized KIF1A with reducing agents (Fig. 4). This result is similar to oxidized myosin, where motor activity can be rescued by treatment with glutathione (22). Oxidative modifications that are known to be reversible by reducing agents include disulfide bond formation, S-sulfenilation, and S-nitrosylation (13). The intradimer cross-linking shown by the nonreducing gels in Figure 7 clearly point to intradimer disulfide bond formation. Previous work on both kinesin-1 and KIF1A have shown that intradimer cross-linking of the two motor domains inhibits motility (33, 39). Thus, the ability of reducing agents to partially restore the fraction of processive motors (Fig. 4*B*) is consistent with reversal of intradimer crosslinking. The ability of reducing agents to partially restore the single-molecule velocity (Fig. 4*C*) is consistent with oxidation causing reversible S-sulfenilation and S-nitrosylation of cysteines, although it does not rule out other mechanisms.

Treatment of KIF1A with 100 mM H_2_O_2_ led to irreversible damage of both WT KIF1A and KIF1A lacking any cysteines in its motor domain. Thus, oxidation targets residues beyond just cysteines and could include methionine, histidine, tryptophan, tyrosine, proline, arginine, and lysine (40). Cysteine mutations were not benign and, in particular, removal of multiple cysteines in the motor domain significantly diminished motility. However, when normalized to their untreated motility, the relative decrease in the velocity of the cysteine mutuants at moderate [H_2_O_2_] was reduced relative to WT (Fig. 8*F*). Previous work has shown that certain redox-switchable proteins can be activated and inactivated through formation of a single disulfide bond (41, 42). The current data argue against a redox switch mechanism of this type in KIF1A. Furthermore, the inability of cysteine removal to protect against oxidative damage suggests that removal or protection of cysteines in KIF1A is not a promising approach for reducing oxidative damage of KIF1A that may be associated with aging or neurodegenerative disease.

### Limitations and implications

The oxidative stress imposed in this study was acute (30 min) and strong (up to 100 mM H_2_O_2_), whereas oxidative stress in cells is more moderate but chronic. During chronic exposure, the buildup of irreversible modifications over time is a concern; however these effects are balanced by cellular ROS scavenging enzymes, glutathione, and antioxidants that help to protect intracellular proteins from oxidative damage (43). H_2_O_2_ exposure is a common approach for inducing oxidative stress *in vitro*, but both the specific modifications and the extent of oxidation are expected to differ under *in vivo* conditions.

Previous work showed that exposing neurons in culture to H_2_O_2_ result in diminished axonal transport, axonal fragmentation, and diminished neural function (25). Oxidative stress was shown to increase the microtubule catastrophe frequency (21) and cause structural damage to microtubules in cardiac myocytes (20). Interestingly, we found that when microtubules were treated with H_2_O_2_ levels that strongly modified KIF1A, there was no change in the resulting KIF1A motility. This result suggests that impacts of oxidative stress on axonal transport are more likely due to effects on the motors than on the filaments. However, oxidative damage of microtubules could also contribute to disease pathogenesis in a number of ways, and it is possible that the taxol stabilization used in our assay diminished the impacts of oxidative stress; hence, this conclusion is still speculative. Alzheimer's disease is associated with hyperphosphorylated tau aggregates, which are hypothesized to inhibit kinesin-based motility (44, 45, 46). Disulfide bond formation in tau has been linked to enhanced tau accumulation and toxicity in a tauopathy animal model, suggesting that oxidative stress may affect tau (14). One promising study showed that chemically modifying cysteine residues in tau reduced the amount of insoluble tau and enhanced neural function in a tauopathy model (47). Thus, there are multiple targets of oxidative stress that might impact axonal transport. The present study establishes that oxidative stress inhibits KIF1A function, supporting KIF1A as a potential target of oxidative damage in neurodegenerative diseases and aging.

## Experimental procedures

### Protein constructs, purification, and activity quantification

The KIF1A-560-GFP construct consisted of the motor head and neck linker domains (residues 1–368) of *R. norvegicus* KIF1A followed by 216 residues (residues 345–560) from the neck-coil and coil-1 domain of *D melanogaster* KHC, and a C-terminal GFP and His_6_ tag (26). The full sequence is as follows, with cysteine residues (5 in the motor domain, 1 in the coiled-coil, and 2 in GFP) in bold:

MAGASVKVAVRVRPFNSREMSRDSK**C**IIQMSGSTTTIVNPKQPKETPKSFSFDYSYWSHTSPEDINYASQKQVYRDIGEEMLQHAFEGYNV**C**IFAYGQTGAGKSYTMMGKQEKDQQGIIPQL**C**EDLFSRINDTTNDNMSYSVEVSYMEIY**C**ERVRDLLNPKNKGNLRVREHPLLGPYVEDLSKLAVTSYNDIQDLMDSGNKARTVAATNMNETSSRSHAVFNIIFTQKRHDAETNITTEKVSKISLVDLAGSERADSTGAKGTRLKEGANINKSLTTLGKVISALAEMDSGPNKNKKKKKTDFIPYRDSVLTWLLRENLGGNSRTAMVAALSPADINYDETLSTLRYADRAKQIR**C**NAIINEDPNNKLAEEWKRRYEKEKEKNARLKGKVEKLEIELARWRAGETVKAEEQINMEDLMEASTPNLEVEAAQTAAAEAALAAQRTALANMSASVAVNEQARLATE**C**ERLYQQLDDKDEEINQQSQYAEQLKEQVMEQEELIANARREYETLQSEMARIQQENESAKEEVKEVLQALEELTVNYDQKSQEIDNKNKDIDALNEELQQKQSVFNAASTELQQLKDMSMVSKGEELFTGVVPILVELDGDVNGHKFSVSGEGEGDATYGKLTLKFI**C**TTGKLPVPWPTLVTTLTYGVQ**C**FSRYPDHMKQHDFFKSAMPEGYVQERTIFFKDDGNYKTRAEVKFEGDTLVNRIELKGIDFKEDGNILGHKLEYNYNSHNVYIMADKQKNGIKVNFKIRHNIEDGSVQLADHYQQNTPIGDGPVLLPDNHYLSTQSALSKDPNEKRDHMVLLEFVTAAGITLGMDELYKHHHHHH.

The KIF1A-406 construct was identical but lacked KHC residues 407 to 560 and the GFP. Motors were bacterially expressed and purified by nickel column chromatography following published protocols (48, 49). Expression was induced using 0.75 mM isopropyl β-D-1-thiogalactopyranoside and carried out overnight at 21 °C. Bacteria were pelleted, resuspended in lysis buffer (50 mM sodium phosphate, 40 mM imidazole, 300 mM NaCl_2_, 10 μM leupeptin, 10 μM pepstatin, 100 μM MgATP, 5 mM βME, pH 7.0.) and sonicated, and the lysate was ultracentrifuged at 183,000g for 35 min at 4 °C. The supernatant was incubated with Ni agarose beads for 1 h, the slurry was added to a gravity column and washed with 20 ml lysis buffer. Proteins were eluted with 5 ml of elution buffer (50 mM sodium phosphate, 300 mM imidazole, 300 mM NaCl_2_, 10 μM MgATP, 4.67 mM DTT, pH 7.0). Fractions with the most obvious green fluorescence were combined, 10% glycerol was added as a cryoprotectant, the solution was aliquoted into smaller volumes, and the aliquots were flash-frozen on liquid nitrogen and stored at −80 °C. KIF1A-560-GFP concentration was quantified using GFP absorption at 488 nm.

### H_2_O_2_ treatment

Oxidation of KIF1A was carried out by combining 2.2 μM KIF1A in elution buffer with different concentrations of H_2_O_2_ diluted from a stock solution of 30% w/w (8.82 M) aqueous hydrogen peroxide (Sigma-Aldrich, H1009–100 Ml). The reaction was carried out at room temperature for 30 min, a duration that was previously shown to achieve full oxidative modification of myosin (22). Following incubation, the sample was placed back on ice and used immediately after for a motility assay. Of note, there was some residual DTT in the motor samples from the purification and storage, which could have partially quenched the H_2_O_2_, particularly at sub-mM concentrations of H_2_O_2_ where no effects were seen. However, the motility changes at 1 mM H_2_O_2_ argue against it having a significant impact on 1, 10, and 100 mM H_2_O_2_ treatments.

Oxidation of microtubules was performed inside the flow chamber where microtubules were fixed on the glass slide and stabilized with 10 μM taxol. A solution of 10 mM H_2_O_2_ aqueous hydrogen peroxide was flushed inside the flow chamber and allowed to sit for 30 min. The flow chamber was then flushed with 20 μl wash solution (2 mg/ml casein, 2 mM MgATP, and 10 μM taxol), and the KIF1A motors flushed in and imaged.

### Gliding assay

Bovine brain microtubules were polymerized by combining 32 μM tubulin (purified as previously described (48)) with 5% dimethyl sulfoxide, 4 mM MgCl_2_, and 1 mM GTP in BRB80 buffer (80 mM K-Pipes, 1 mM MgCl_2_, 1 mM EGTA, pH 6.9) and incubating for 30 min at 37 °C. Polymerized microtubules were stabilized by diluting eight fold into BRB80 containing 10 μM taxol and kept at room temperature.

Flow cells were constructed by adhering double-sided tape to a glass slide to form a channel, placing a glass coverslide on top, and heating the flow cell at 100 °C for 30 s to allow the tape to melt and resolidify. To perform the gliding assay, the following solutions (all in BRB80) were flushed in the flow cell channel: (1) 20 μl of 0.042 mg/ml anti-His-tag antibody (Invitrogen Thermo Fisher Scientific, product number: 4A12E4) with 5 min incubation, (2) 20 μl wash solution (2 mg/ml casein, 2 mM MgATP, and 10 μM taxol), (3) 20 μl of 100 nM KIF1A motors in 2 mg/ml casein, (4) 20 μl wash solution, (5) 20 μl of 0.080 μM microtubules in 2 mg/ml casein and 1 mM MgATP or MgADP. Gliding microtubules were visualized as previously described (30) under the Nikon TE-2000 TIRF microscope using interference reflection microscopy (50) using a blue (440 nm) LED (pE-300white, CoolLED) at 1.25% power. The LED was attached to the fluorescent line and the illumination numerical aperture was optimized to achieve the maximum signal-to-noise, following Mahamdeh et al (50). Videos were of five frames per seconds. The movies were analyzed with FIJI ImageJ to measure gliding velocity.

### Single-molecule fluorescence tracking

Single-molecule tracking of GFP-labeled KIF1A-560 was performed on a Nikon TE2000 TIRF microscope at 25 °C, as described previously (27, 28, 29). Flow cells were prepared and the following solutions flowed in (all in BRB80 buffer with 5 min incubation for each): 0.5 mg/ml casein, 40 nM full-length rigor kinesin (27), and taxol-stabilized microtubules (80 nM final [tubulin]), polymerized from a 1:20 ratio of Cy5-labeled (GE Healthcare) and unlabeled tubulin. Finally, 300 pM motors (diluted from a 2.2 μM stock) were introduced in motility solution (BRB80 containing 2 mg/ml casein, 20 mM glucose, glucose oxidase, catalase, 10 μM Taxol, and 2 mM MgATP) and imaged with an Andor EMCCD camera at 5 fps. The motility solution did not include βME so as not to interfere with the oxidative state of oxidized KIF1A. Also note that the motor dilution reduced the H_2_O_2_ concentration nearly 7000-fold such that the maximum H_2_O_2_ concentration in motility assays was 13 μM (100 mM treatment). Videos were manually analyzed using FIJI ImageJ to determine the run length, velocity, and dwell times. In the ADP dwell time assays, some trials contained hexokinase to hydrolyze any contaminating ATP. In single-molecule motility assays, 200 mM βME was used to reverse oxidative damage, whereas crosslinking analysis by gel electrophoresis used 200 mM DTT to reverse oxidative damage. This choice was based on previous protocols (23, 48) and the two reducing agents were assumed to be interchangeable at these concentrations, although this was not rigorously tested.

### Sodium dodecyl sulfate-polyacrylamide gel electrophoresis (SDS-PAGE)

For gel analysis, NuPAGE LDS Sample Buffer (4X) was added to the protein samples and the samples heated to 100 °C for 5 min for denaturation. Protein samples (10 μl of ∼1 μM motors) were run on NuPAGE Bis-Tris Mini Protein Gels 4 to 12% at 120 V for 1 h, stained with Coomassie blue for 1 h, and then destained with distilled water. Photographs of gels were imported into FIJI (ImageJ), cropped, and the intensity adjusted uniformly across the image using linear contrast adjustment to maximize visibility of bands.

## Data availability

Complete data for all figures are publicly available at: http://doi.org/10.26207/64f0-dq40.

## Conflict of interest

The authors declare that they have no conflicts of interest with the contents of this article.
